# Supplementary material for: Tead1a Initiates Transcriptional Priming Through the TEAD1a/YAP‐Notch1‐Spi1/Cebpα Axis to Promote Neutrophil Fate
Source: Adv Sci (Weinh). 2025 Aug 28;12(41):e05441. doi: 10.1002/advs.202505441 (PMC12591127; doi:10.1002/advs.202505441)
Supplement: Supplementary file 4 — Supplementary Dataset [file ADVS-12-e05441-s004.zip › 3-6.zip!/3-6/02-6.cluster/cluster17/Enrich/ego_CC.pdf]

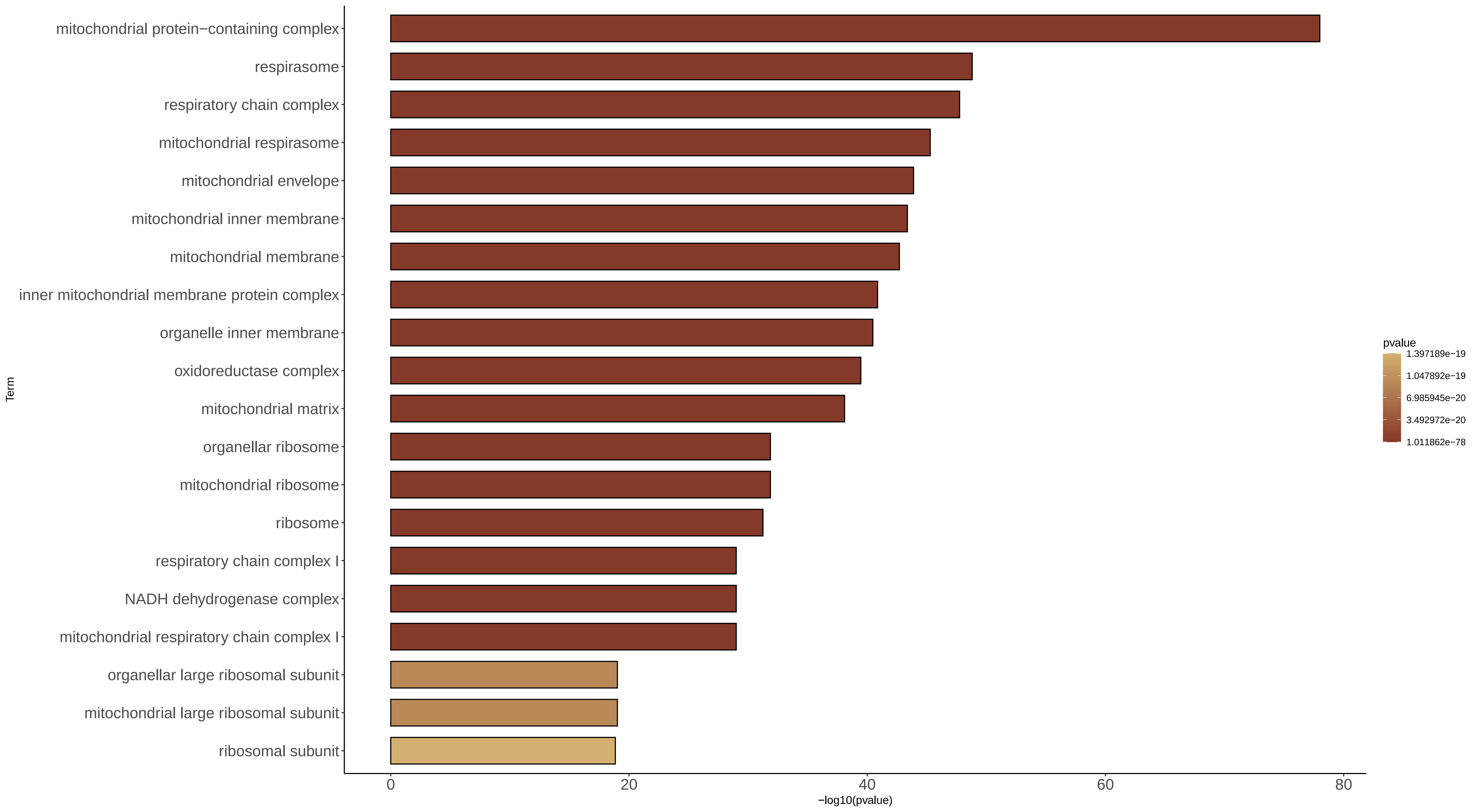
mitochondrial protein−containing complex
respirasome
respiratory chain complex
mitochondrial respirasome
mitochondrial envelope
mitochondrial inner membrane
mitochondrial membrane
inner mitochondrial membrane protein complex
organelle inner membrane
oxidoreductase complex
mitochondrial matrix
organellar ribosome
mitochondrial ribosome
ribosome
respiratory chain complex I
NADH dehydrogenase complex
mitochondrial respiratory chain complex I
organellar large ribosomal subunit
mitochondrial large ribosomal subunit
ribosomal subunit
Term
0
20
40
60
80
−log10(pvalue)
pvalue
1.397189e−19
1.047892e−19
6.985945e−20
3.492972e−20
1.011862e−78
